# Chronic diseases of aging in an evolutionary context

**DOI:** 10.1093/emph/eoaa013

**Published:** 2020-05-08

**Authors:** Benjamin C Trumble, India Schneider-Crease

**Affiliations:** e1 School of Human Evolution and Social Change, Arizona State University, Tempe, AZ, USA; e2 Center for Evolution and Medicine, Arizona State University, Tempe, AZ, USA; e3 School of Life Sciences, Arizona State University, Tempe, AZ, USA

## DEFINITION AND BACKGROUND

As we age, we become increasingly likely to face cardiovascular diseases, cognitive decline, benign prostatic hyperplasia and other chronic diseases of aging (CDA). Research on the drivers of these diseases will be vital in the coming half century, over which the US Centers for Disease Control and Prevention (CDC) projects that the elderly population (>65 years) in the USA will double to reach 98 million. Because humans lived as subsistence populations for 99% of our history (i.e. as hunter-gatherers or horticulturalists), examining the incidence of CDA in contemporary subsistence populations will contribute an essential evolutionary perspective to treatment and prevention initiatives. The long-held assumption that people in subsistence populations do not live long enough to experience CDA is based on the low life expectancy at birth of these populations (21–37 years). However, this metric is skewed by infant mortality, which is ∼35 times higher in subsistence populations than in industrialized ones. A better measure of longevity is the modal age of death for adults; across all subsistence populations, the modal age of death is between 68–78 years and 20–30% of people live past that age [1]. Many people in subsistence populations thus live long enough to experience CDA, but research summarizing patterns of these diseases within and across subsistence populations remains scant.

## EXAMPLES IN CLINICAL MEDICINE AND PUBLIC HEALTH

**Figure 1.**
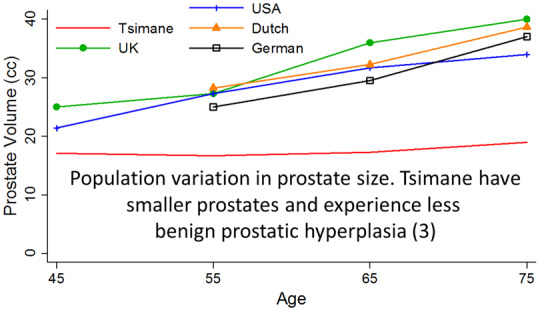
Population variation in prostate size. Tsimane have smaller prostates and experience less benign prostatic hyperplasia [3]

**Figure 2.**
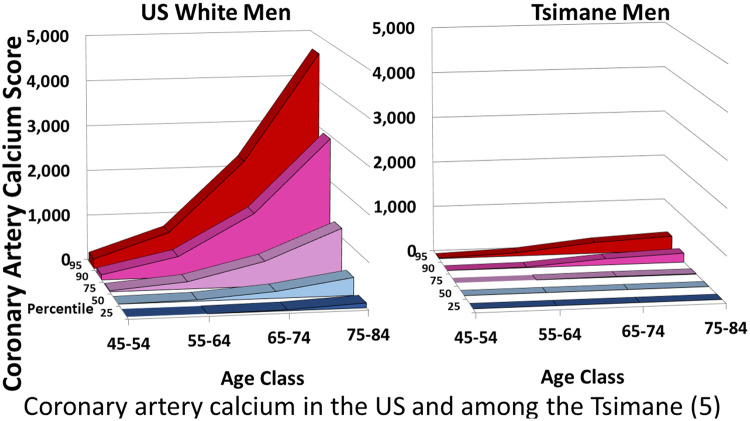
Coronary artery calcium in the USA and among the Tsimane [5]

The available data suggest that CDA occur at lower rates in subsistence populations compared to industrialized populations, and that this phenomenon is tied to physiological, behavioral and environmental differences that modulate risk. For example, lower sex-specific cancer rates among subsistence populations may result from lower lifetime exposure to reproductive hormones [2]. Women in subsistence populations have 4-fold fewer lifetime menstrual cycles due to high parity and long breastfeeding duration. Combined with lower absolute levels of ovarian hormones, the reduction in exposure to reproductive hormones may explain the significantly lower breast cancer rates in these populations compared to US women [2]. Similarly, testosterone is lower in men in subsistence populations, as are rates of testosterone-linked conditions including benign prostatic hyperplasia [3] (Fig. 1). Lower rates of obesity in subsistence populations [4]—in which obtaining enough calories to become obese is difficult and in which obesity would impede foraging—likely modulate risk for CDA. Indeed, obesity-related diseases, such as diabetes [5], polycystic ovary syndrome [6] and cardiovascular diseases including hypertension, stroke and coronary artery calcification [4, 5, 7] are rare in these populations (Fig. 2). Contributing to lower obesity rates—and, by extension, lower CDA rates—are dietary differences, low pollution exposure and high pathogen exposure. Immunomodulation from pathogens and pollution increases lipid and glucose metabolism, subsequently decreasing adipose deposition [5, 7]. Industrialized urban life is evolutionarily novel, and physiological, behavioral and environmental mismatches may be responsible for the high prevalence of many chronic diseases. Expanding research within and among subsistence populations will provide critical evolutionary context for what we see in the clinic today.
